# Oxidative stress mediated by gyrophoric acid from the lichen *Umbilicaria hirsuta* affected apoptosis and stress/survival pathways in HeLa cells

**DOI:** 10.1186/s12906-019-2631-4

**Published:** 2019-08-19

**Authors:** Michal Goga, Martin Kello, Maria Vilkova, Klaudia Petrova, Martin Backor, Wolfram Adlassnig, Ingeborg Lang

**Affiliations:** 1 Core Facility Cell Imaging and Ultrastructure Research, University of Vienna, Althanstrasse 14, 1090 Vienna, Austria; 2 Department of Botany, Institute of Biology and Ecology, Faculty of Science, Pavol Jozef Šafárik University, Mánesova 23, 041 67 Košice, Slovakia; 3 Department of Pharmacology, Faculty of Medicine, Pavol Jozef Šafárik University, Trieda SNP 1, 040 11 Košice, Slovakia; 4 Department of NMR Spectroscopy, Institute of Chemistry, Faculty of Science, Pavol Jozef Šafárik University, Moyzesova 11, 040 11 Košice, Slovakia

**Keywords:** Gyrophoric acid, Cervical cancer, Apoptosis, Oxidative stress, p38MAPK, Erk1/2, Akt

## Abstract

**Background:**

Lichens produce a huge diversity of bioactive compounds with several biological effects. Gyrophoric acid (GA) is found in high concentrations in the common lichen *Umbilicaria hirsuta*, however evidence for biological activity was limited to anti-proliferative activity described on several cancer cell lines.

**Methods:**

We developed and validated a new protocol for GA isolation, resulting in a high yield of highly pure GA (validated by HPLC and NMR) in an easy and time saving manner. Anti-proliferative and pro-apoptotic activity, oxygen radicals formation and stress/survival proteins activity changes was study by flow cytometry.

**Results:**

The highly purified GA showed anti-proliferative activity against HeLa (human cervix carcinoma) and other tumor cells. Moreover, GA threated cells showed a significant increase in caspase-3 activation followed by PARP cleavage, PS externalization and cell cycle changes mediated by oxidative stress. Production of oxygen radicals led to DNA damage and changes in stress/survival pathways activation.

**Conclusions:**

GA treatment on HeLa cells clearly indicates ROS production and apoptosis as form of occurred cell death. Moreover, DNA damage and changing activity of stress/survival proteins as p38MAPK, Erk1/2 and Akt mediated by GA treatment confirm pro-apoptotic potential. The pharmacological potential of *U. hirsuta* derived GA is discussed.

**Electronic supplementary material:**

The online version of this article (10.1186/s12906-019-2631-4) contains supplementary material, which is available to authorized users.

## Background

Cancer belongs to the global causes of mortality worldwide. Natural products as bioactive compounds from microorganisms, plants and marine organisms served in fight with cancer. Lichens represent chemically important symbiotic organisms of fungi (mycobiont) and algae/cyanobacteria (photobiont) which produce a various secondary metabolites. Approximately 1000 secondary metabolites were discovered so far and they are specific for lichens [1, 2]. Secondary metabolites are classified by their biosynthetic origins and chemical structures.

A wide spectrum of biological activity of secondary metabolites is known so far. Dibenzofurans, depsides and depsidones, naphthoquinones, anthraquinones, xanthones and some other specific class compounds showed promising anticancer potential [3–6]. One of the most studied lichen polyphenolic compounds with high biological activity, including antiproliferative effect, are depsides and depsidones [7–9]. Bačkorová et al. [4] showed antiproliferative activity and induction of apoptosis mediated by well-known depside atranorin in wide spectrum of cancer cell lines. Promising results showed also treatments with another depside as lobaric acid [10], protolichesterinic acid [11], olivetoric acid [12] and physodic acids [13] with high anti-cancer potential.

Gyrophoric acid (GA) is a characteristic compound of the lichen genus *Umbilicaria*. It is known as good ultraviolet filter in lichen populations. As was shown, GA effectively avoids cytotoxic and apoptotic activity of UVB in dose-dependent manner in irradiated HaCaT cells [14]. Besides photoprotective activity, GA showed relatively strong antimicrobial effect against several bacteria and fungi among which were human pathogens [15]. Moreover, antioxidant properties of gyrophoric acid were confirmed by DPPH radical-scavenging activity [16].

Anticancer activity of *Umbilicaria* species were confirmed by screening test [17]. Antiproliferative effect of gyrophoric acid on cancer cell lines were demonstrated in several studies [4, 18, 19]. Bačkorová et al. [19] demonstrated that 200 μM dose of gyrophoric acid led to significant decrease of mitochondrial membrane potential in ovarian cancer cells A2780 after 24 h long exposure but not in HT-29 colon adenocarcinoma cells. The same dose significantly increased the proportion of Annexin V positive cells after 24 h long exposure in A2780 while in HT-29 after 72 h. Production of ROS was observed only in HT-29 cells after 3 and 6 h, whereas in A2780 cells were not affected. Furthermore, western blot analysis showed GA-mediated alteration of apoptotic proteins p53, Bcl-2, Bax in A2780 cells and proteins p53, Bcl-xL, Bax and p38 in HT-29 cells. Similarly, in study Cardile et al. [13], gyrophoric acid significantly inhibited cell growth and affected the expression of Bcl-2, Bax and Hsp70 proteins but only on higher concentration in A375 melanoma cancer cells.

Despite the above mentioned works, there is still a lack of information about apoptotic mechanisms influenced by GA treatment. For this reason, in our experiments we focused on influence of GA treatment on modulation stress/survival pathways p38 MAPK, Erk1/2, Akt and possible pro-oxidant and genotoxic activity.

## Methods

### Lichen material

*Umbilicaria hirsuta* (Sw. Ex Westr.) was collected from extrusive igneous volcanic rocks Sninský kameň (48°55’46”N 22°11′23”E) in Vihorlat Mountains (Prešov, Slovakia), during November, 2016. *U. hirsuta* was collected and determined by Dr. Goga. Lichen specimen was deposited in the herbarium of P.J. Šafárik University in Košice (KO). The lichen thalli of *U. hirsuta* were wetted with distilled water and carefully removed from the rock surface.

### Preparation of lichen extract

The lichen material was rinsing with distilled water and air-dried at room temperature (26 °C) for 48 h. Extraction of lichen material was performed in falcon tubes. 5 g/DW of *U. hirsuta* was extracted with 50 ml of water free acetone for 24 h in order to reduce extraction of intracellular compounds. During this time, the falcon tube was vortexed four times. The extract was filtered by nylon sifter (pore size 42 μm). Extraction was repeated two times, pooled, and acetone was evaporated by rotar evaporator. After cooling the residue to 4 °C the residue was rinsed by methanol (2–5 ml) slightly, and supernatant and pellet were separated. In order to maximalise the yield, the methanol phase was centrifuged for 20 min at 14000 rpm. The pellet was pooled with residue of evaporation. This process was repeated until no pellet was formed.

### High-performance liquid chromatography (HPLC)

The white powder, resulting from the extraction procedure was analysed by semi preparative HPLC with DAD detection (Agilent Technologies 1260 Infinity device). A 7 μm Kromasil SGX C18 column was used. Mobile phase A (5% acetonitrile + 1% (v/v) trifluoracetic acid) and mobile phase B (80% acetonitrile) were in isocratic program with a flow rate of 0.7 mL min^− 1^: 0 min 50% A and 50% B; 25 min 0% A and 100% B; 30 min 50% A and 50% B. For quantitative analysis of GA, the wavelength of 270 nm was used.

### Nuclear magnetic resonance (NMR) spectroscopy

The structure of the compound was verified by NMR spectra at room temperature on NMR spectrometer Varian VNMRS600 (PaloAlto, CA, USA) operating at 599.868 MHz for ^1^H and 150.836 MHz for ^13^C. Spectra were recorded in DMSO-d_6_. The 2D NOESY, Heteronuclear single quantum correlation (gHSQC) and Heteronuclear Multiple Bond Correlation (gHMBC) methods were employed.

### Cell cultures

The human cancer cell line HeLa (human cervix carcinoma), MCF-7 (human breast adenocarcinoma), A549 (human lung adenocarcinoma) and HDF (human dermal fibroblasts) were obtained from ATCC- American Type Culture Collection (Manassas, VA, USA). HeLa cells were cultured in RPMI 1640 medium (Biosera, Kansas City, MO, USA) and MCF-7, A549 and HDF cells in a DMEM medium with sodium pyruvate (GE Healthcare, Piscataway, NJ, USA). Growth medium was supplemented with a 10% fetal bovine serum (FBS), penicillin (100 IU/ml) and streptomycin (100 μg/ml) (all Invitrogen, Carlsbad, CA, USA). All cell lines were maintained in standard cancer cell culture conditions (5% CO_2_ in humidified air at 37 °C). Cell viability before all experiments was greater than 95%.

### MTS cell proliferation/viability assay

Cell viability and proliferation was determined using standard MTS assay (Promega, Madison, WI, USA). Cells were seeded at a density of 1 × 10^4^ cells/well in 96-well plates. Twenty four hours after cell seeding, different concentrations (150–350 μM) of the GA and cisplatin (Cis-Pt 13 μM) were directly applied. NAC/GA experimental groups were pre-treated with N-Acetyl-L-cysteine (NAC c = 2 mM) and T/GA groups with Trolox (c = 100 μM) (all Sigma Aldrich, St. Louis, MO, USA) for 1 h before GA was added. After 72 h of incubation, 10 μl of MTS were added to each well. After an additional 2 h, cell proliferation was evaluated by measuring of the absorbance at wavelength 490 nm using the automated Cytation™ 3 Cell Imaging Multi-Mode Reader (Biotek, Winooski, VT, USA). Absorbance of control wells was taken as 1.0 = 100%, and the results were expressed as a fold/percentage of untreated control. IC50 values were calculated from MTS analyses.

### Cell cycle analysis

Floating and adherent HeLa cells (1 × 10^6^) were harvested together 24, 48 and 72 h after GA treatment (c = 150 μM). NAC/GA experimental groups were pre-treated with N-Acetyl-L-cysteine (NAC c = 2 mM) for 1 h before GA was added. Complete cell population was washed in phosphate- buffered saline (PBS), fixed in cold 70% ethanol and kept at + 4 °C overnight. Before analyses, fixed cells were washed in PBS and stained in PBS solution (500 μl) containing 0.2% Triton X-100, 0.5 mg/ml ribonuclease A and 0.025 mg/ml propidium iodide (all Sigma Aldrich). Samples were incubated for 30 min at room temperature in the dark. The DNA contents of the stained cells, representative for each phase of cell cycle, were analysed using a flow cytometer BD FACSCalibur (BD Biosciences, San Jose, CA, USA).

### Apoptosis detection via Annexin V/PI staining

Phosphatidyl serine (PS), a phospholipid, is normally localized on the inner surface of the lipid bilayer of the plasma membrane. Externalization of PS on the other side of plasmatic membrane can be detected by the Annexin V-FITC conjugate. Annexin V staining therefore acts as a marker of programmed cell death. For apoptosis detection, floating and adherent HeLa cells (1 × 10^6^) were harvested 24, 48 and 72 h after GA treatment (c = 150 μM). NAC/GA experimental groups were pre-treated with N-Acetyl-L-cysteine (NAC c = 2 mM) for 1 h before GA was added. Complete cell population was washed in PBS and stained using Annexin-V-FLUOS Staining Kit (Roche Diagnostics, Mannheim, Germany) for 15 min at room temperature in the dark followed by incubation with propidium iodide (PI) and analyses by flow cytometer (BD FACSCalibur).

### Detection of active caspase 3 and poly ADP ribose polymerase (PARP) cleavage

Caspases are proteolytic enzymes playing a crucial role in controlling cell death. Activation of executioner caspases (such as caspase 3) subsequently impacts the main structural proteins and activates other enzymes, leading to apoptosis. The changes in caspase 3 activation and PARP cleavage were analysed with FCM using Active Caspase-3 PE Mab and Cleaved-PARP (Asp214) XP® Rabbit mAb (PE Conjugate) (Cell Signaling Technology, Danvers, MA, USA). The cells were harvested 24, 48 and 72 h after GA treatment (c = 150 μM). NAC/GA experimental groups were pre-treated with N-Acetyl-L-cysteine (NAC c = 2 mM) for 1 h before GA was added. Cell population was stained with phycoerythrin (PE) conjugated antibody and incubated for 30 min at room temperature in the dark. The cells were then washed twice with PBS, resuspended in 500 μM of the total volume, and analysed (1 × 10^4^ cell per sample). Fluorescence was detected with 585/42 (FL-2) optical filter by flow cytometer (BD FACSCalibur).

### Detection of mitochondrial membrane potential (MMP)

Mitochondria are described as key factors in controlling apoptosis. Disruption of MMP was analysed with FCM using 0.1 μM TMRE (Molecular Probes, Eugene, OR, USA) staining. After 30 min of incubation at room temperature in the dark the stained cells were then washed twice with PBS, resuspended and analysed (1 × 10^4^ cells per sample). Fluorescence was detected with 585/42 (FL-2) optical filter by flow cytometer (BD FACSCalibur).

### Measurement of superoxide anions and reactive oxygen species (ROS)

Oxygen radicals are produce intracellularly and detected with FCM analysis using MitoSOX™Red mitochondrial superoxide indicator (Thermo Fisher, Waltham, MA, USA) or dihydrorhodamine-123 (DHR-123, Fluka), which reacts with intracellular hydrogen peroxide (ROS). The cells treated with gyrophoric acid were harvested, washed two times in PBS and resuspended in PBS. DHR-123 was added at a final concentration 0.2 μM and MitoSOX red at 5 μM. The samples were then incubated for 15 min in dark and after incubation were placed on ice. Fluorescence was detected with 530/30 (FL-1) resp. 585/42 (FL-2) optical filter by flow cytometer (BD FACSCalibur). Forward and side scatters were used to gate the viable populations of cells.

### DNA damage detection

The cells treated with gyrophoric acid were harvested, washed two times in PBS and resuspended in PBS. Changes of guanine oxidation were analysed by Anti-Oxoguanine 8 antibody (Abcam, Cambridge, UK) on BD FACSCalibur flow cytometer.

### Stress/survival proteins activity

Flow cytometry analyses of phosphorylated proteins involved in stress/survival pathways were performed. The cells treated with gyrophoric acid were harvested, washed two times in PBS and stained 30 min by Phospho-p44/42 MAPK (Erk1/2) (Thr202/Tyr204) (E10) Mouse mAb (Alexa Fluor® 488 Conjugate), Phospho-Akt (Ser473) (D9E) XP® Rabbit mAb (PE Conjugate) or Phospho-p38 MAPK (Thr180/Tyr182) (3D7) Rabbit mAb (PE Conjugate) (All Cell Signaling). Fluorescences were detected by BD FACSCalibur flow cytometer.

### Statistical analysis

Results are expressed as arithmetic mean ± SD. Statistical analyses included one-way ANOVA followed by Bonferroni multiple comparisons test. Differences were considered significant when *p* values were smaller than 0.05. Throughout this paper * indicates *p* < 0.05, ** *p* < 0.01, *** *p* < 0.001. *n* = 3 for all experiments. Spearman’s Rank-Order Correlation was done using SPSS Statistics software (IBM, Armonk, NY, USA).

## Results

### Identification of lichen secondary metabolites by HPLC and NMR

The HPLC chromatogram of the acetone extract of *U. hirsuta* is shown in Additional file 1 (A, B). The supernatant fraction dissolved in methanol showed acetone (1), an unknown compound (2) and GA (3) (Additional file 1A), whereas the pellet dissolved in acetone showed acetone (1) and GA (2) only (Additional file 1B). The purity of GA was 98, 2%. The UV spectrum of isolated GA recorded from the HPLC chromatogram and the structure recorded from NMR spectrum are shown in Additional file 2.

The ^1^H NMR spectrum of GA displayed three pairs of meta coupled doublets at δ_H_ 6.68 and 6.67 ppm (*J* 2.1 Hz), δ_H_ 6.64 and 6.62 ppm (*J* 2.1 Hz), δ_H_ 6.24 and 6.23 ppm (*J* 2.2 Hz), assigned to the H-3′ and H-5′ protons of the three aromatic rings A, B and C, respectively (Additional file 3). Besides the signals of methyl groups (δ_H_ 2.37, 2.37 and 2.35 ppm), we detected three signals attributed to hydroxyl groups at δ_H_ 10.00, 10.30 and 10.47 ppm, in the ^1^H NMR spectrum.

The evidence for the localization of the methyl and hydroxyl groups on the aromatic rings was provided by the Heteronuclear Multiple Bond Correlation (HMBC) experiment (Additional file 5). HMBC spectra showed HMBC correlations between methyl group protons and carbon atoms C-1, C-5 and C-6, and HMBC correlations between hydroxyl proton (δ_H_ 10.47 ppm) bound to carbon atom C-2’A (δ_C_ 156.2 ppm) and C-1’A (δ_C_ 138.0 ppm) and C-3’A (δ_C_ 107.2 ppm). Next, HMBC correlations between hydroxyl proton at δ_H_ 10.30 ppm and carbon atoms C-1’C (δ_C_ 140.2 ppm) and C-3’C (δ_C_ 100.5 ppm) and hydroxyl proton at δ_H_ 10.00 ppm and carbon atoms C-3’C (δ_C_ 100.5 ppm) and C-5’C (δ_C_ 109.8 ppm) allowed to distinguished the terminal aromatic ring (Additional file 4). It was not possible to determine quaternary carboxyl carbons in the HMBC experiment Additional file 5, as these chemical shifts may be mutually interchangeable. However the direct determination of carbon and hydrogen (HSQC) could be confirmed (Additional file 6).

### Analyses of cell proliferation after GA treatment

**Fig. 1:**
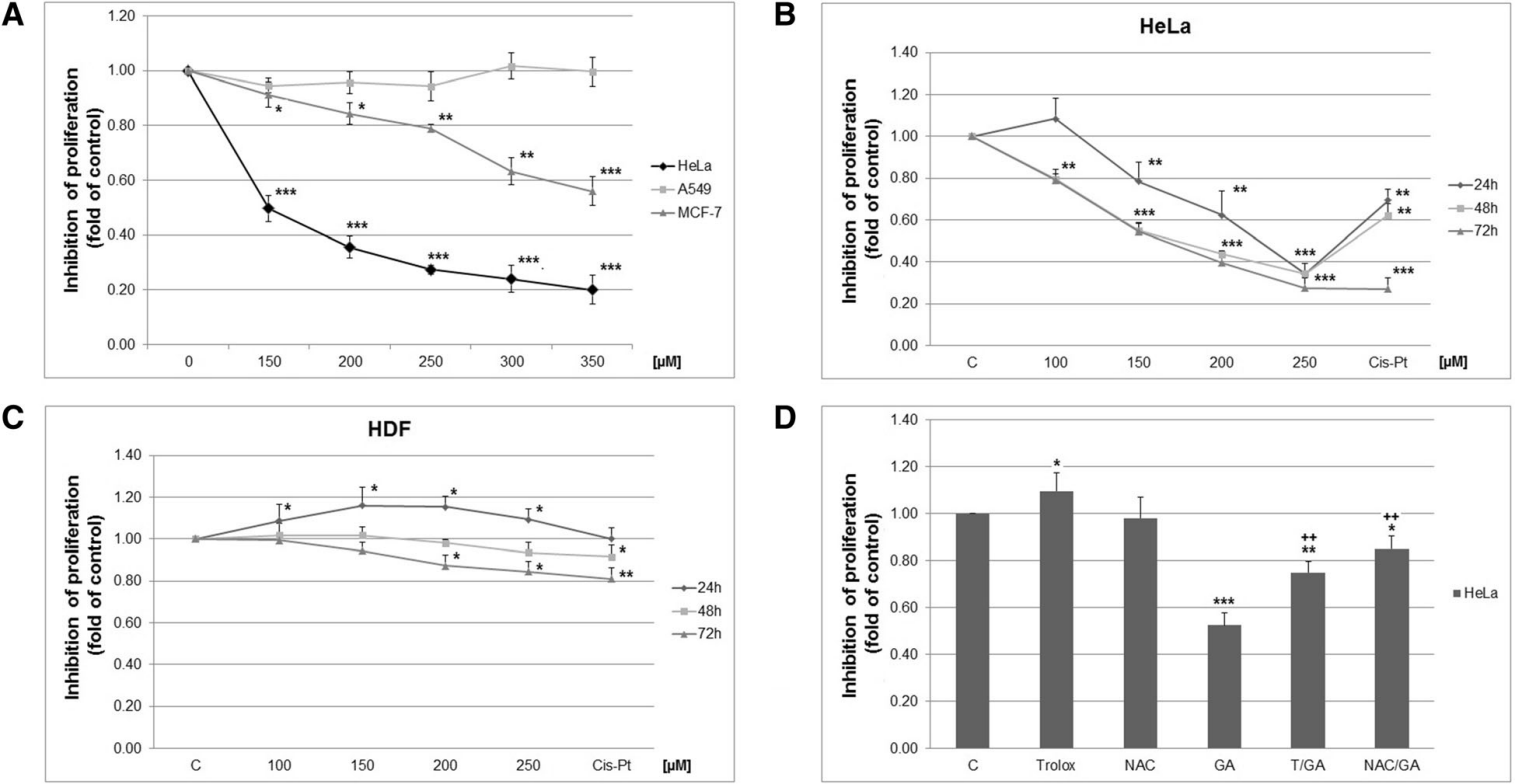
**a** Effect of GA (72 h) on HeLa, MCF-7 and A549 cells proliferation; Time and dose-dependent analyses of HeLa (**b**) and HDF (**c**) cells proliferation using MTS assay. **d** HeLa cells proliferation affected by NAC/Trolox pre-treatment on 72 h. Data are presented as mean ± SD from 3 independent experiments. Significance: * *P* < 0.05, ** *P* < 0.01, *** *P* < 0.001 versus untreated cells (control); ^++^ *P* < 0.01, versus GA

IC_50_ values of the colorimetric MTS assay on several cancer cell lines are presented in Table 1. GA exhibited the most significant inhibitory effects on the growth of HeLa cells (Fig. 1a) reducing proliferation capacity with IC50 value of 145.42 (± 4.82) μmol.l^− 1^. Other tested cell lines displayed weaker effect on cell proliferation. GA-treatment in HeLa cells showed time- and dose-dependent inhibition of proliferation (Fig. 1b). Human dermal fibroblasts were used as healthy cell model with weaker response only at 72 h and higher concentrations used. Compared with cisplatin (drug control), GA-treatment of HDF cells showed less toxicity to healthy cell population (Fig. 1c). Based on these results, further experiments were performed with the most sensitive cancer cell line, i.e. HeLa, using a final concentration c = 150 μmol.l^− 1^.

**Table 1:** The IC50 (μM) of tested compounds in different cell lines after 72 h incubation. Data is presented as a mean ± SD of two independent experimental determinations performed in triplicate

Compound	Cancer Cell Lines - IC_50_ (μM)		
	A549	MCF-7	HeLa
**GA**	> 500	384.14 ± 1.92	145.42 ± 4.82

### Oxidative stress induction and DNA damage after GA treatment

**Fig. 2:**
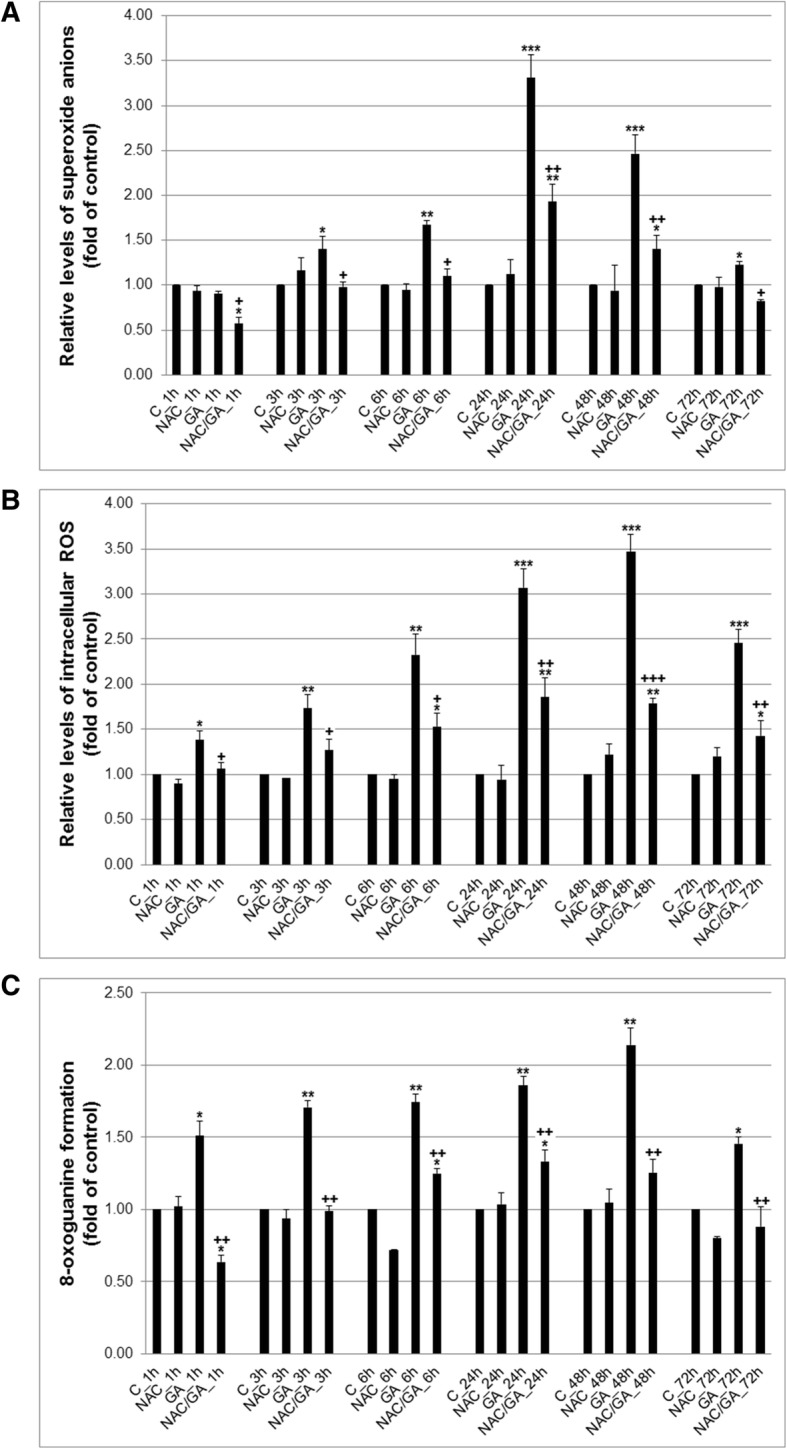
Effect of GA alone or after NAC pre-treatment on superoxide anion generation (**a**), ROS levels (**b**) and DNA damage induction (**c**) in HeLa cells. Data were obtained from three independent flow cytometry experiments after 1–72 h GA treatment. Significance: * *P* < 0.05, ** *P* < 0.01, *** *P* < 0.001 versus untreated cells (control); ^+^ *P* < 0.05, ^++^ *P* < 0.01, ^+++^ *P* < 0.001 versus GA

Programmed cell death after several treatments can be mediated and triggered by several mechanisms included oxidative stress. In our experiment, we realised to study changes in redox potential of GA to induce oxidative stress. In general, oxygen radicals are involved in organelle and DNA damage and act as initiator of apoptotic process. Two species of oxygen radicals were analysed: superoxide anions and ROS (peroxides intermediates). Potent antioxidant N-Acetyl-L-cysteine (NAC) was used in all followed experiments to elucidate activity of GA-mediated oxygen species in anti-proliferative and pro-apoptotic mechanisms. NAC was able to scavenger both O_2_^−^ and ROS peroxide species. We noticed both oxygen species formation shortly after GA administration from 3 h, with culmination at 24 h (O_2_^−^) resp. 48 h (peroxides), which contributed probably on several followed pro-apoptotic processes (Fig. 2a, b).

**Fig. 3:**
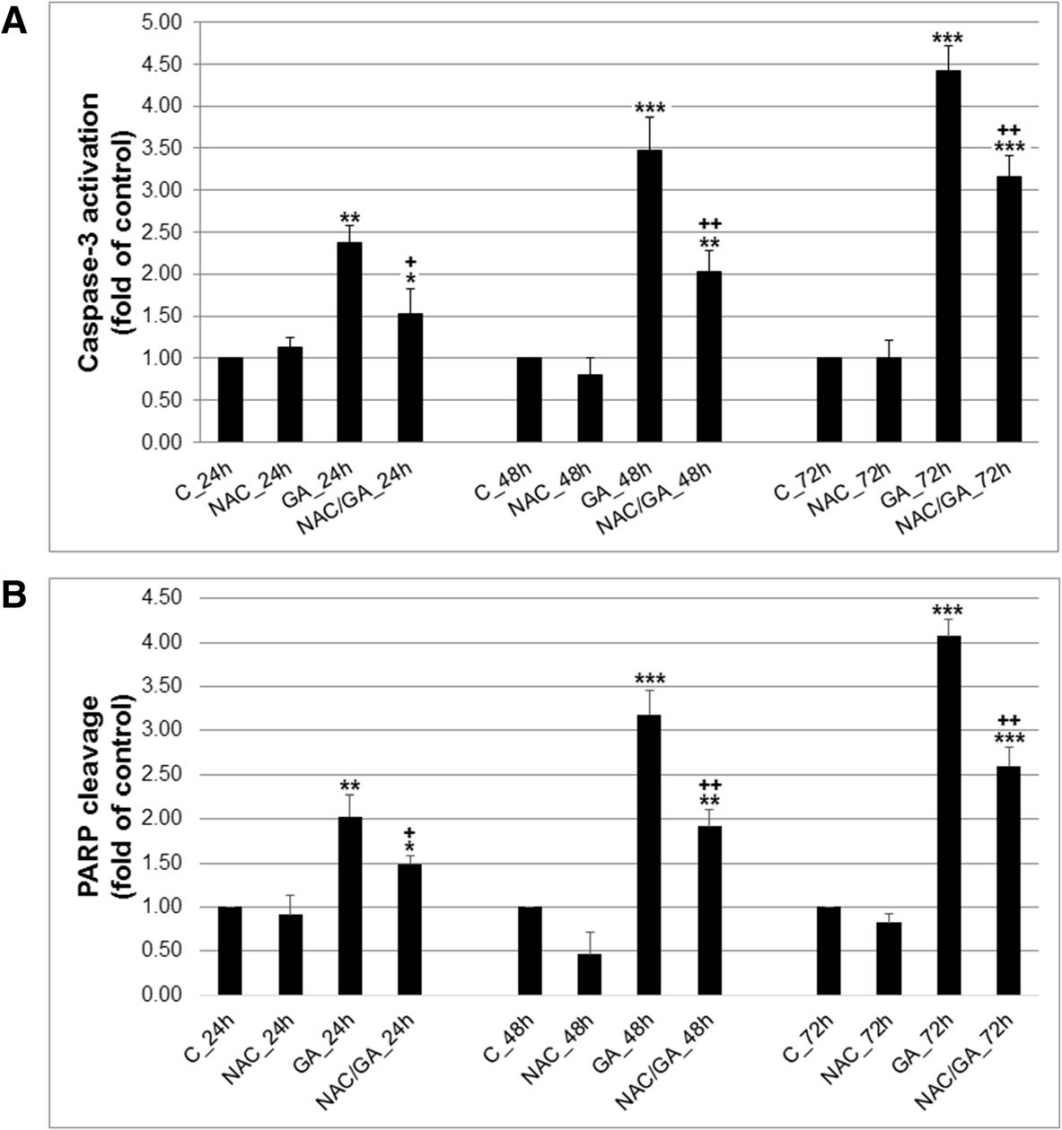
**a** Effect of GA alone or after NAC pre-treatment on caspase-3 activity. **b** Effect of GA alone or after NAC pre-treatment on PARP cleavage. Data represents mean ± SD from 3 independent experiments. Significance: * *P* < 0.05, ** *P* < 0.01, *** *P* < 0.001 versus untreated cells (control); ^+^ *P* < 0.05, ^++^ *P* < 0.01, versus GA

Oxidative stress mediated by all kind of oxygen and nitrogen radicals usually leads to DNA damage. To analyse impact of GA-mediated oxidative stress on DNA, 8-oxoguanine formation as biomarker of DNA damage was analysed. We noticed that DNA damage increased by time to 48 h. Moreover, pre-treatment with NAC (by scavenger activity) significantly protect HeLa cells from DNA damage at all analyses time-point (Fig. 3c).

### Caspase 3 activation and cleavage PARP after GA treatment

To support findings that GA treatment has cytotoxic effect on Hela cells, we tested execution phase of apoptosis via caspase-3 activation and PARP cleavage. We marked that caspase-3 activity (Fig. 3a) significantly increased after 24 h and persisted to 72 h after GA treatment. Moreover, caspase 3 activity was correlated strongly (Spearman’s *p* = 0.90***) with PARP cleavage (Fig. 3b), which increased in the same manner after 24 h. These findings confirmed time- and caspase-dependent occurrence of cell death after GA treatment. Partial involvement of GA-mediated oxidative stress in caspase-dependent apoptosis was confirmed by use of NAC. Oxygen species scavenging clearly reduce caspase-3 activation followed by reduced PARP cleavage compare to GA treatment. ROS activity correlated both with caspase-3 ((Spearman’s *p* = 0.61*) and PARP results (Spearman’s *p* = 0.66 *).

### Mitochondrial membrane potential (MMP) changes

**Fig. 4:**
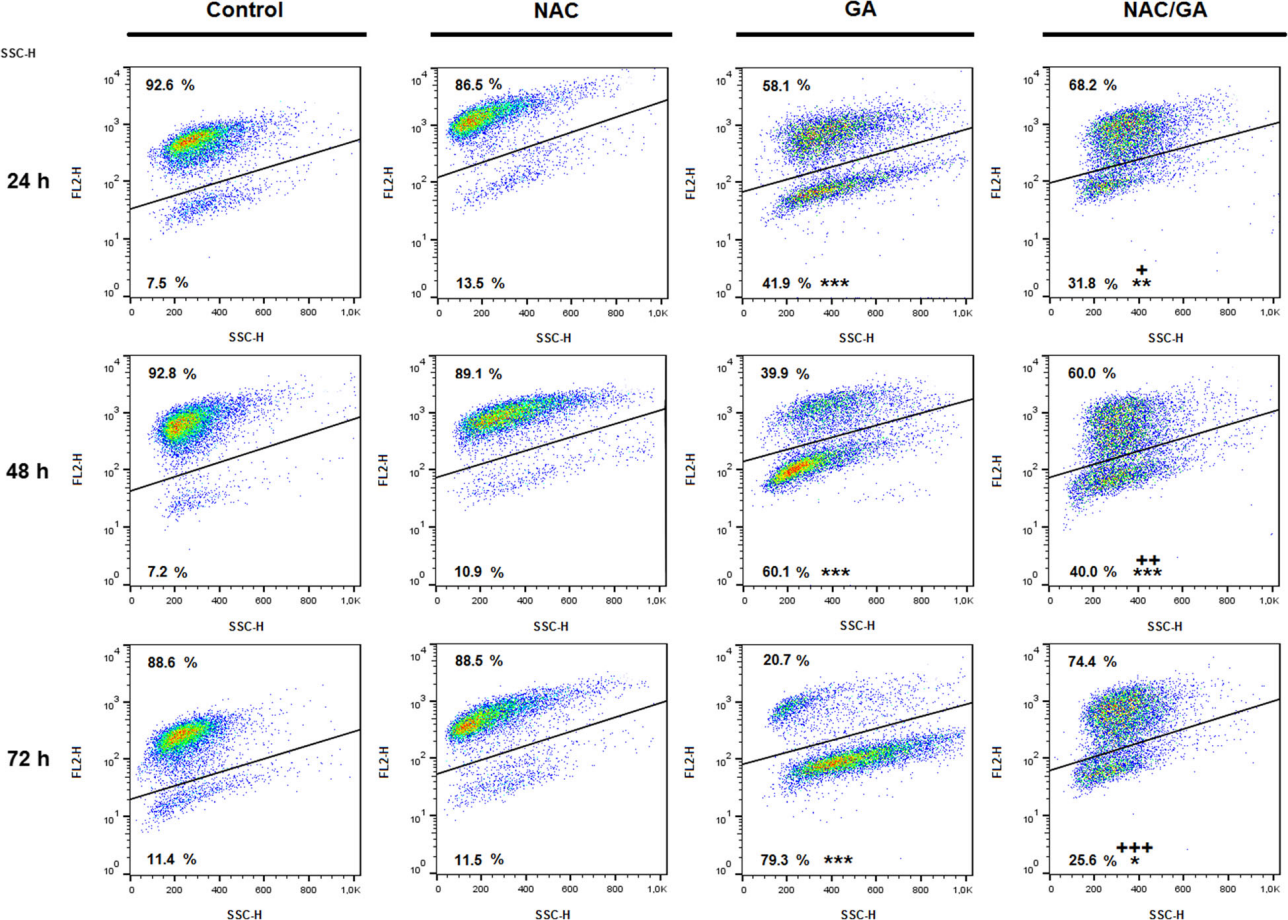
Representative dot-plot diagram of MMP changes after GA treatment alone or after NAC pre-treatment. Data were obtained from three independent experiments and significant differences were marked as * *P* < 0.05, ** *P* < 0.01, *** *P* < 0.001 versus untreated cells (control); ^+^ *P* < 0.05, ^++^ *P* < 0.01, ^+++^ *P* < 0.001 versus GA

Mitochondria represent the key organelle affected by several extra- or intracellular stimuli. Mitochondrial membrane damage (mostly by oxygen species) and MMP changes represent very early indicator of mitochondrial dysfunction leading to apoptosis. As shown on Fig. 4, GA treatment increased significantly population of cells with dissipated MMP after 24, 48 and 72 h. Moreover, protection of cells by NAC scavenger activity led to decreasing of cell population with lower MMP. Is obvious that oxidative stress play important role in apoptosis mediated by GA. These finding are also in correlation with cytotoxic effect and induction of apoptosis after GA treatment alone or after pre-treatment of HeLa cells with NAC.

### GA- mediated changes in cell cycle of HeLa cells population

**Fig. 5:**
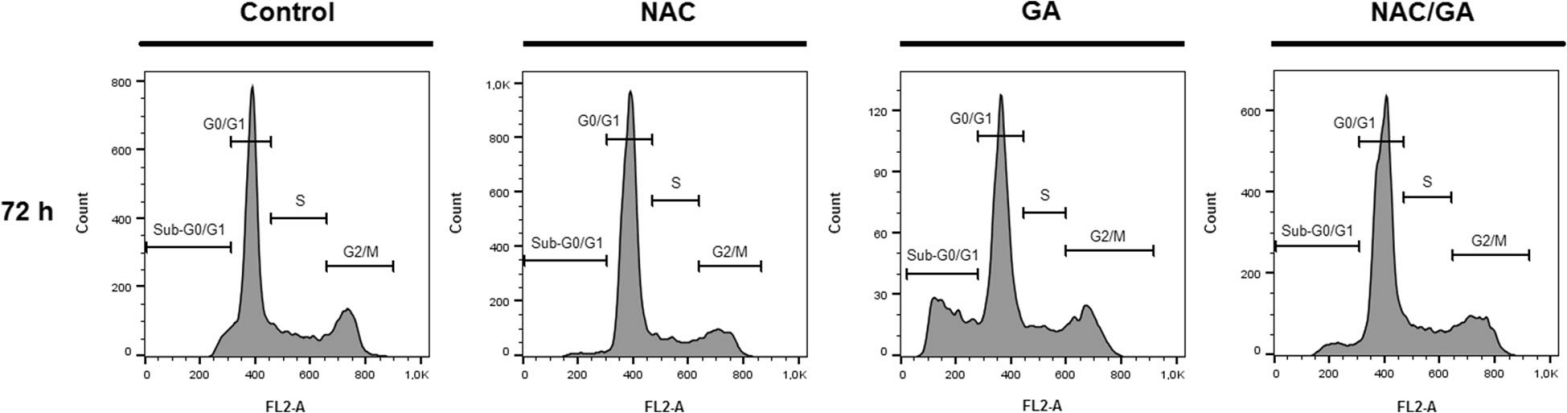
Representative histograms of cell cycle distribution after GA treatment alone or after NAC pre-treatment

To determine whether GA-mediated cell death is related to cell cycle arrest, a flow cytometry cell cycle analysis was performed. As shown on Table 2 and Fig. 5, HeLa cells exposed to GA treatment exhibited significant increase of cells with sub-G0/G1 DNA content (the marker of apoptosis) after 24 h treatment that enhances after 48 and 72 h. At the same time, cells in G1 phase significantly decreased after 24 h exposure to GA. However, no cell cycle arrest occurred after GA treatment in any time point. NAC pre-treatment, similar as in caspase-3 and PARP analyses, was able partially decrease occurrence of apoptotic HeLa cells after GA treatment.

**Table 2:** Flow cytometric analysis of cell cycle distribution in HeLa cells treated with GA (in %). The results are presented from 3 independent experiments as mean ± SD; significantly different, * *P* < 0.05, ** *P* < 0.01 versus untreated cells (control); ^+^ *P* < 0.05 versus GA. Sub-G0/G1 fraction of cells identified as apoptotic population

Treatment	Time (h)	sub-G_0_/G_1_	G_0_/G_1_	S	G_2_/M
Control	24	1.50 ± 0.33	57.70 ± 1.54	20.80 ± 1.35	20.00 ± 1.11
	48	1.80 ± 0.71	64.40 ± 1.88	17.40 ± 0.89	16.30 ± 0.61
	72	2.80 ± 0.25	69.20 ± 1.77	14.50 ± 1.20	13.60 ± 0.55
NAC	24	2.90 ± 1.10	58.90 ± 1.41	18.80 ± 0.99	19.40 ± 1.22
	48	3.10 ± 0.77	67.50 ± 2.32	13.80 ± 0.57	15.60 ± 0.88
	72	2.70 ± 0.43	70.40 ± 1.19	12.40 ± 0.66	14.50 ± 0.67
GA	24	8.00 ± 1.30^*^	50.00 ± 2.11^*^	20.00 ± 1.23	22.00 ± 1.19
	48	16.20 ± 1.20^**^	46.50 ± 2.75^**^	18.50 ± 1.70	18.90 ± 1.44
	72	20.90 ± 1.55^**^	47.90 ± 1.35^**^	16.50 ± 1.79	14.80 ± 1.30
NAC/GA	24	4.60 ± 0.21	60.70 ± 1.14^+^	18.20 ± 0.73	16.50 ± 1.13
	48	7.30 ± 0.76^*,+^	60.90 ± 2.10^+^	14.30 ± 1.30	17.50 ± 1.90
	72	5.50 ± 1.25^+^	60.70 ± 1.93^*,+^	15.60 ± 0.56	18.20 ± 0.33

### Apoptosis detection via externalization of PS after GA treatment

Typical mark of apoptotic cell death initiation is externalisation of phosphatidyl serine on the outer side of plasmatic membrane. In our experiments, GA induced significant increase in cellular apoptosis (early stage, An+/PI-) of HeLa cells and PS externalization already after 24 h treatment with persistence after 48 and 72 h (Table 3). Moreover, we observed an increase in cells positively stained with both Annexin V and PI (late apoptotic events or cell death, An+/PI+) particularly after 24, 48 and 72 h. Moreover, NAC pre-treatment significantly reduce both early and late apoptotic cells population after GA treatment followed by concomitant increase of An−/PI- Live cells population in all analysed time-points.

**Table 3:** Annexin V/PI flow cytometry analysis of apoptosis occurrence in HeLa cells after GA treatment alone and after NAC pre-treatment (in %). The results are presented from 3 independent experiments. Significantly different, *P < 0.05, **P < 0.01, ***P < 0.001 versus untreated cells (control); ^+^*P* < 0.05, ^++^*P* < 0.01 versus GA

Treatment	Time (h)	An^−^/PI^−^ Live cells	An^+^/PI^−^ Early apoptotic	An^+^/PI^+^ Late apoptotic and death
Control	24	90.90 ± 0.65	2.50 ± 0.16	6.57 ± 0.42
	48	89.60 ± 1.11	2.60 ± 0.59	7.79 ± 1.23
	72	89.50 ± 1.99	3.17 ± 0.60	7.32 ± 0.81
NAC	24	89.10 ± 1.66	3.50 ± 0.44	7.35 ± 0,66
	48	92.30 ± 1.82	1.88 ± 0.33	5.77 ± 0.28
	72	94.80 ± 1.43	1.68 ± 0.19	3.56 ± 0.37
GA	24	52.80 ± 2.45^**^	22.70 ± 1.00^**^	24.53 ± 3.29^*^
	48	30.30 ± 2.99^***^	31.30 ± 2.66^**^	38.40 ± 2.45^**^
	72	28.40 ± 1.75^***^	21.80 ± 2.12^**^	49.78 ± 3.33^***^
NAC/GA	24	72.90 ± 1.77^*,++^	16.00 ± 0.90^*,+^	11.11 ± 1.71^*,+^
	48	68.30 ± 2.20^**,++^	13.60 ± 1.59^*,+^	18.14 ± 1.54^*,++^
	72	67.40 ± 3.19^**,++^	11.30 ± 0.69^*,+^	21.25 ± 2.34^*,++^

### Effect of GA treatment on MAPK, Erk and Akt activation

**Fig. 6:**
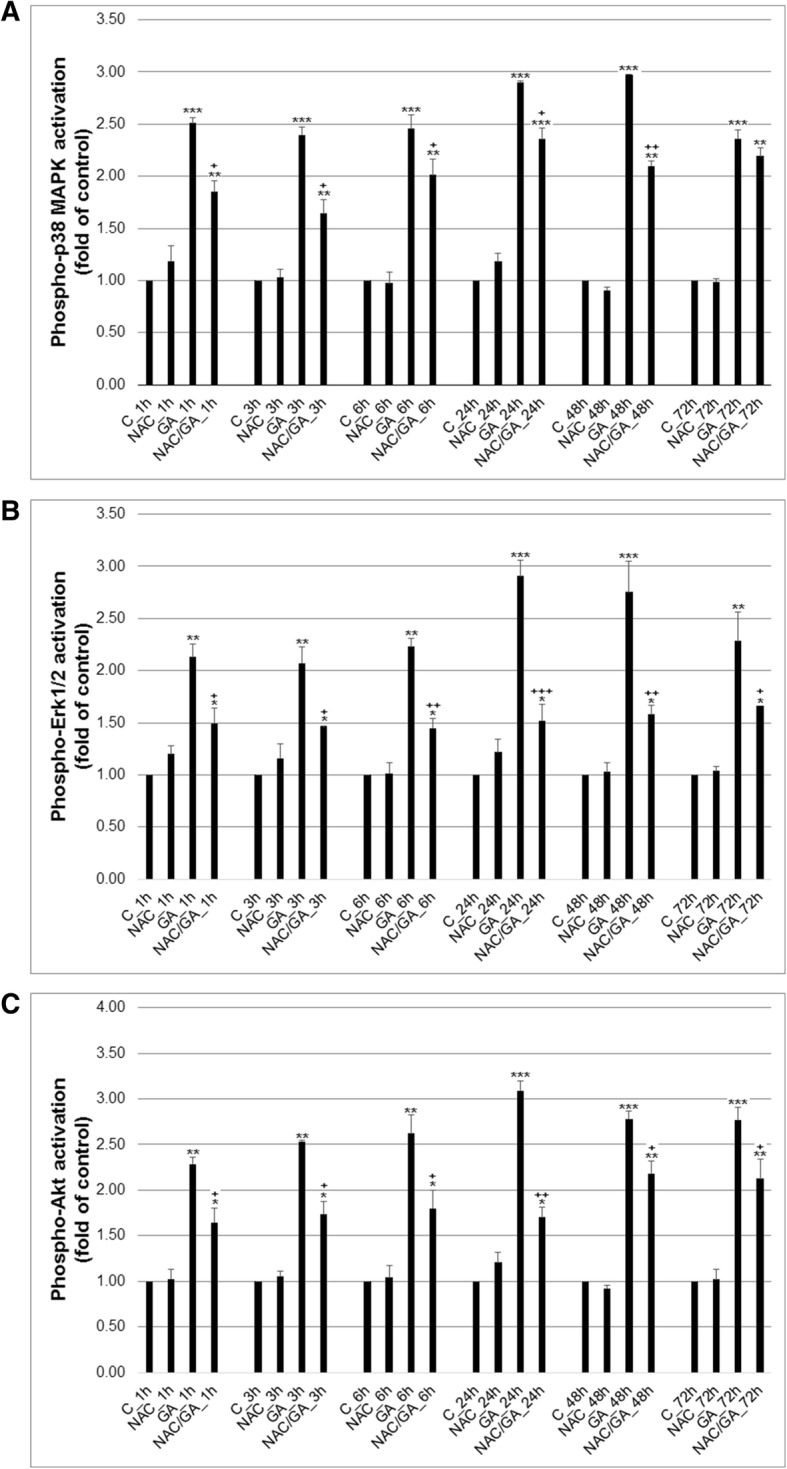
Effect of GA alone or after NAC pre-treatment on phosphorylation status of signal proteins p38 MAPK (**a**), Erk 1/2 (**b**) and Akt (**c**) in Hela cells. Data were obtained from three independent flow cytometry experiments after 1–72 h GA treatment. Significance: * *P* < 0.05, ** *P* < 0.01, *** *P* < 0.001 versus untreated cells (control); ^+^ *P* < 0.05, ^++^ *P* < 0.01, ^+++^ *P* < 0.001 versus GA

Several proteins are cross-linked in stress/survival pathways included members of MAPK family (JNK, p38 MAPK, Erk) and Akt protein. To study effect of GA treatment on stress/survival pathways proteins activation, we analysed phosphorylation status of p38 MAPK, Erk 1/2 and Akt proteins. We noticed that GA significantly increased phosphorylation of all tested proteins (Fig. 6) soon after 1 h treatment and with maximum at 24 (Erk, Akt) resp. 48 h (p38 MAPK). On the other hand, NAC pre-treatment partially inhibited phosphorylation of all proteins after exposure HeLa cells to GA, suggested oxidative stress mediated anti-survival changes in stress/survival pathways machinery.

## Discussion

So far more than 1000 acidic metabolites are known from lichens [20, 21]. In spite of the anticancer and antiproliferative activity of some of these compounds [2] the low concentrations within a tiny thallus (5–10% of dry weight) together with laborious isolation prevent pharmaceutic use [22].

*U. hirsuta* contains GA as a main metabolite accompanied only by small amounts of lecanoric acid [23]. By using and novel, simple and economic procedure for extracting and purifying GA, we achieved a purity of 98, 2%. After 48 h of extraction in acetone, we obtained 45.5 mg/1.0226 g of DW representing 4, 45% of GA, thereby significantly improving the method described by Solhaung and Gauslaa [24]. Since *U. hirsuta* is found frequently on the appropriate substrate and is no endangered collection of the lichen and extraction of GA for pharmacological properties appears feasible. Furthermore GA is a cortical metabolite which occurs as crystals on the surface of the hyphae. Therefore homogenization of thallus is not necessary, which avoids the extraction of numerous other compounds. Modification of GA appears feasible therefore opening a wide field for production of tailor-made derivates.

Previous experiments [4] find moderate evidence of anti-proliferative activity of GA. If highly purified GA isolated by our method was used, cytotoxicity for Hela cells was improved to IC_50_ = 145.42 ± 4.82 vs. > 200 μM. Using MTS tests, we observed cytotoxic effect of GA against three different cell lines. Through HeLa cells were most sensitive and therefore used for further experiments; both our experiments and literature data [1, 4, 25] indicate activity against a very broad spectrum of carcinoma cells in dose-dependent manner.

The mechanism of GA-mediated programmed cell death was completely enigmatic yet. Our results provide strong evidence on multiple scales, that apoptosis plays a key role in the activity of GA. This mechanism has already been described for other lichen metabolites including usnic acid, atranorin, diffractaic acid as well as vulpinic acid [4, 19, 26, 27]. Upon exposure to GA caspase-3 activation, PARP cleavage, oxygen radicals production, MMP dissipation, phosphatidyl serin externalisation and cell cycle exhibited consistently significant changes favouring apoptosis. All these parameters are well known as apoptotic markers [28, 29], therefore we can conclude that apoptosis at least contributes to the anti-proliferative, pro-apoptotic effect of GA, possibly accompanied by membrane damage as suggested by Gupta et al. [30]. Onset of apoptosis was observed after 24 h, which is in accordance with anti-proliferative agents, where reaction time of 24 h or more is common [19]. No cell cycle arrest was observed, indicating a direct induction of apoptosis as described by Sun et al. [31]. Moreover, we observed a time-dependent significant increase in the number of apoptotic HeLa cells as evidenced by Annexin V/PI staining and PARP cleavage. Furthermore, Annexin-V staining showed that the treatment of GA led to an increase of apoptotic cells after 24 h (early phase) and the rapid increase after 48 h and 72 h (in late phase) supported our hypothesis that GA directly induces apoptosis and cell death. These findings are also in accordance with PARP cleavage that increased soon after 24 h of GA treatment and resulted in strong cleavage after 72 h.

In addition, the collapse of mitochondrial membrane potential is the early step in the apoptotic cascade [32]. A rapid collapse of ΔΨm is typically observed in apoptosis induced by anti-proliferative compounds. Our data showed that treatment with GA leads to a collapse of mitochondrial transmembrane potential in a time-dependent manner.

It is clear that GA treatment induce apoptosis of HeLa cells with all common markers (morphology changes, DNA fragmentation, PS externalisation, MMP dissipation, etc...) presented. Apoptosis induction is in general triggered by damaging effects of tested substances, by anti−/pro-oxidant properties and modulation of stress/survival/apoptotic pathways. Oxidative stress and affection of redox balance followed by DNA damage of tumour cells are one of often described mechanisms of plenty natural substances in cancer research [33–36]. As was described in recent past, some lichen secondary metabolites, for example, usnic acid [37] olivetoric, physodic and psoromic acid [12], exert in vitro pro-apoptotic activity through oxidative stress induction and DNA damage. Based on the mentioned facts, we tested possible pro-oxidant and genotoxic activity of GA treatment. Moreover, to confirm direct association of oxygen radicals in GA-mediated DNA damage and apoptosis, we used NAC natural common antioxidant in these experiments. As our analyses confirmed, GA-treatment, similar as above mentioned published data, induced production and accumulation of oxygen radicals (superoxide anion and peroxides) with concomitant DNA oxidation and damage in HeLa cells. In addition, pre-treatment of HeLa cells with antioxidant NAC led to partial reduction of oxygen radicals generation and prevention of DNA oxidation during GA treatment. In general, protection of HeLa cells by antioxidant NAC led to partial lowered oxidative stress and DNA damage, reduction of apoptotic cells occurrence, inhibition of caspase-3 activation and PARP cleavage. Furthermore, as followed analyses showed, GA-mediated oxidative stress induced modulation of stress/survival/apoptotic pathways p38 MAPK, Erk 1/2 and Akt. To this date no data were published about GA mechanisms and involvement of these pathways in GA-mediated apoptosis in HeLa cells. Otherwise, Chen et al. [38] suggested that cytotoxicity of usnic acid may result from Akt/mTOR- and MAPK-mediated pathways. And Backorova et al. [19] showed that p38 MAPK kinase phosphorylation associated with apoptosis increased in the presence of parietin in A2780 cells or in presence of atranorin and usnic acids in HT-29 cells. In general, phosphorylation and activation of p38 MAPK is involved in apoptotic processes, while phosphorylation of Erk 1/2 and Akt is involved in cell survival. In our experiments we have observed increased phosphorylation of all tested proteins after GA-treatment of HeLa cells and partial inhibition of phosphorylation after NAC pre-treatment. Protection of HeLa cells with antioxidant clearly showed that GA-mediated production of oxygen radicals is directly associated with p38 MAPK, Erk 1/2 and Akt phosphorylation status, thus is involved in apoptosis and survival. Although the ERK, signalling is usually associated with cell proliferation, controversially, several studies showed that Erk 1/2 phosphorylation lead to initiation of apoptosis and cell death [39, 40], which is in agreement with our results.

## Conclusion

- GA can be efficiently isolated in a simple and economical manner from *U. hirsuta* by using a novel extractive procedure
- Induction of apoptosis is the most probable explanation for the anti-proliferative effects of GA
- Pro-apoptotic potential of GA is mediated by oxidative burst and DNA damage.
- GA treatment influence stress/survival pathways (p38MAPK, Erk1/2, Akt).
- Derivates of GA could be used in order to achieve specific pharmaceutic effects

## Additional files

Additional file 1: Chromatogram of GA supernatant dissolved in methanol. (PNG 173 kb)

Additional file 2: UV-spectrum from HPLC and the structure of gyrophoric acid. (JPG 463 kb)

Additional file 3: ^1^H-NMR Spectrum of gyrophoric acid from acetone extract of the lichen *Umbilicaria hirsuta. (BMP 2422 kb)*

Additional file 4: ^13^C-NMR Spectrum of gyrophoric acid from acetone extract of the lichen *Umbilicaria hirsuta. (BMP 2422 kb)*

Additional file 5: Selected HMBC correlations in gyrophoric acid. (PNG 16 kb)

Additional file 6: HSQC spectrum of gyrophoric acid (BMP 2422 kb)

## Data Availability

The datasets used and/or analysed during the current study are available from the corresponding author on reasonable request. Plant materials are available by Dr. Michal Goga.

## References

[1] Stanojković T, Ranković B (2015). Investigations of lichen secondary metabolites with potential anticancer activity. Lichen secondary metabolites: bioactive properties and pharmaceutical potential.

[2] Goga M, Elečko J, Marcinčinová M, Ručová D, Bačkorová M, Bačkor M, Merillon J-M, Ramawat KG (2018). Lichen metabolites: an overview of some secondary metabolites and their biological potential. Co-evolution of secondary metabolites.

[3] Babula P, Adam V, Havel L, Kizek R (2009). Noteworthy secondary metabolites naphthoquinones - their occurrence, pharmacological properties and analysis. Curr Pharm Anal.

[4] Backorova M, Backor M, Mikes J, Jendzelovsky R, Fedorocko P (2011). Variable responses of different human cancer cells to the lichen compounds parietin, atranorin, usnic acid and gyrophoric acid. Toxicol In Vitro.

[5] Felczykowska A, Pastuszak-Skrzypczak A, Pawlik A, Bogucka K, Herman-Antosiewicz A, Guzow-Krzemińska B (2017). Antibacterial and anticancer activities of acetone extracts from in vitro cultured lichen-forming fungi. BMC Complement Altern Med.

[6] Ranković BR, Kosanić MM, Stanojković TP (2011). Antioxidant, antimicrobial and anticancer activity of the lichens Cladonia furcata, Lecanora atra and Lecanora muralis. BMC Complement Altern Med.

[7] Russo A, Caggia S, Piovano M, Garbarino J, Cardile V (2012). Effect of vicanicin and protolichesterinic acid on human prostate cancer cells: role of Hsp70 protein. Chem Biol Interact.

[8] Ranković B, Kosanić M, Manojlović N, Rančić A, Stanojković T (2014). Chemical composition of Hypogymnia physodes lichen and biological activities of some its major metabolites. Med Chem Res.

[9] Bessadóttir M, Skúladóttir EÁ, Gowan S, Eccles S, Ómarsdóttir S, Ögmundsdóttir HM (2014). Effects of anti-proliferative lichen metabolite, protolichesterinic acid on fatty acid synthase, cell signalling and drug response in breast cancer cells. Phytomedicine.

[10] Emsen B, Aslan A, Turkez H, Joughi A, Kaya A (2018). The anti-cancer efficacies of diffractaic, lobaric, and usnic acid: In vitro inhibition of glioma. J Cancer Res Ther.

[11] Brisdelli F, Perilli M, Sellitri D, Bellio P, Bozzi A, Amicosante G, Nicoletti M, Piovano M, Celenza G (2016). Protolichesterinic acid enhances doxorubicin-induced apoptosis in HeLa cells in vitro. Life Sci.

[12] Emsen B, Aslan A, Togar B, Turkez H (2016). In vitro antitumor activities of the lichen compounds olivetoric, physodic and psoromic acid in rat neuron and glioblastoma cells. Pharm Biol.

[13] Cardile V, Graziano ACE, Avola R, Piovano M, Russo A (2017). Potential anticancer activity of lichen secondary metabolite physodic acid. Chem Biol Interact.

[14] Varol M, Türk A, Candan M, Tay T, Koparal AT (2016). Photoprotective activity of Vulpinic and Gyrophoric acids toward ultraviolet B-induced damage in human keratinocytes. Phytother Res.

[15] Ranković B, Mišić M, Sukdolak S (2008). The antimicrobial activity of substances derived from the lichens Physcia aipolia, Umbilicaria polyphylla, Parmelia caperata and Hypogymnia physodes. World J Microbiol Biotechnol.

[16] Buçukoglu TZ, Albayrak S, Halici MG, Tay T (2013). Antimicrobial and antioxidant activities of extracts and lichen acids obtained from some Umbilicaria species from Central Anatolia, Turkey. J Food Process Preserv.

[17] Kosanic M, Rankovic B, Stanojkovic T, Vasiljevic P, Manojlovic N (2014). Biological activities and chemical composition of lichens from Serbia. EXCLI J.

[18] Burlando B, Ranzato E, Volante A, Appendino G, Pollastro F, Verotta L (2009). Antiproliferative effects on tumour cells and promotion of keratinocyte wound healing by different lichen compounds. Planta Med.

[19] Backorova M, Jendzelovsky R, Kello M, Backor M, Mikes J, Fedorocko P (2012). Lichen secondary metabolites are responsible for induction of apoptosis in HT-29 and A2780 human cancer cell lines. Toxicol In Vitro.

[20] Molnar K, Farkas E (2010). Current results on biological activities of lichen secondary metabolites: a review. Z Naturforsch C.

[21] Stocker-Worgotter E (2008). Metabolic diversity of lichen-forming ascomycetous fungi: culturing, polyketide and shikimate metabolite production, and PKS genes. Nat Prod Rep.

[22] Oksanen I (2006). Ecological and biotechnological aspects of lichens. Appl Microbiol Biotechnol.

[23] Posner B, Feige GB, Huneck S (1992). Studies on the chemistry of the lichen genus Umbilicaria-Hoffm. Z Naturforsch C.

[24] Solhaug KA, Gauslaa Y (2001). Acetone rinsing - a method for testing ecological and physiological roles of secondary compounds in living lichens. Symbiosis.

[25] Dincsoy AB, Duman DC (2017). Changes in apoptosis-related gene expression profiles in cancer cell lines exposed to usnic acid lichen secondary metabolite. Turk J Biol.

[26] Kilic N, Aras S, Cansaran-Duman D (2018). Determination of Vulpinic acid effect on apoptosis and mRNA expression levels in breast Cancer cell lines. Anti Cancer Agents Med Chem.

[27] Galanty A, Koczurkiewicz P, Wnuk D, Paw M, Karnas E, Podolak I, Wegrzyn M, Borusiewicz M, Madeja Z, Czyz J (2017). Usnic acid and atranorin exert selective cytostatic and anti-invasive effects on human prostate and melanoma cancer cells. Toxicol In Vitro.

[28] Kadam CY, Abhang SA (2016). Apoptosis markers in breast Cancer therapy. Adv Clin Chem.

[29] Sarkar FH, Li Y (2006). Markers of apoptosis. Methods Mol Med.

[30] Gupta VK, Verma S, Gupta S, Singh A, Pal A, Srivastava SK, Srivastava PK, Singh SC, Darokar MP (2012). Membrane-damaging potential of natural L-(−)-usnic acid in Staphylococcus aureus. Eur J Clin Microbiol Infect Dis.

[31] Sun WM, Wang W, Kim J, Keng P, Yang SM, Zhang HS, Liu CM, Okunieff P, Zhang LR (2008). Anti-cancer effect of resveratrol is associated with induction of apoptosis via a mitochondrial pathway alignment. Adv Exp Med Biol.

[32] Wang XD (2001). The expanding role of mitochondria in apoptosis. Genes Dev.

[33] Shafabakhsh R, Asemi Z (2019). Quercetin: a natural compound for ovarian cancer treatment. J Ovarian Res.

[34] Kello M, Kulikova L, Vaskova J, Nagyova A, Mojzis J (2017). Fruit Peel polyphenolic extract-induced apoptosis in human breast Cancer cells is associated with ROS production and modulation of p38MAPK/Erk1/2 and the Akt signaling pathway. Nutr Cancer.

[35] Kello M, Drutovic D, Chripkova M, Pilatova M, Budovska M, Kulikova L, Urdzik P, Mojzis J (2014). ROS-dependent antiproliferative effect of brassinin derivative homobrassinin in human colorectal cancer Caco2 cells. Molecules.

[36] Aborehab NM, Osama N (2019). Effect of Gallic acid in potentiating chemotherapeutic effect of paclitaxel in HeLa cervical cancer cells. Cancer Cell Int.

[37] Araújo AAS, de Melo MGD, Rabelo TK, Nunes PS, Santos SL, Serafini MR, Santos MRV, Quintans-Júnior LJ, Gelain DP (2015). Review of the biological properties and toxicity of usnic acid. Nat Prod Res.

[38] Chen S, Dobrovolsky VN, Liu F, Wu Y, Zhang Z, Mei N, Guo L (2014). The role of autophagy in Usnic acid-induced toxicity in hepatic cells. Toxicol Sci.

[39] Lee WJ, Hsiao M, Chang JL, Yang SF, Tseng TH, Cheng CW, Chow JM, Lin KH, Lin YW, Liu CC (2015). Quercetin induces mitochondrial-derived apoptosis via reactive oxygen species-mediated ERK activation in HL-60 leukemia cells and xenograft. Arch Toxicol.

[40] Lee ER, Kim JY, Kang YJ, Ahn JY, Kim JH, Kim BW, Choi HY, Jeong MY, Cho SG (2006). Interplay between PI3K/Akt and MAPK signaling pathways in DNA-damaging drug-induced apoptosis. Biochim Biophys Acta.

