# Supplementary figures and images for: Oxidative stress mediated by gyrophoric acid from the lichen Umbilicaria hirsuta affected apoptosis and stress/survival pathways in HeLa cells
Source: BMC Complement Altern Med. 2019 Aug 19;19:221. doi: 10.1186/s12906-019-2631-4 (PMC6701105; doi:10.1186/s12906-019-2631-4)

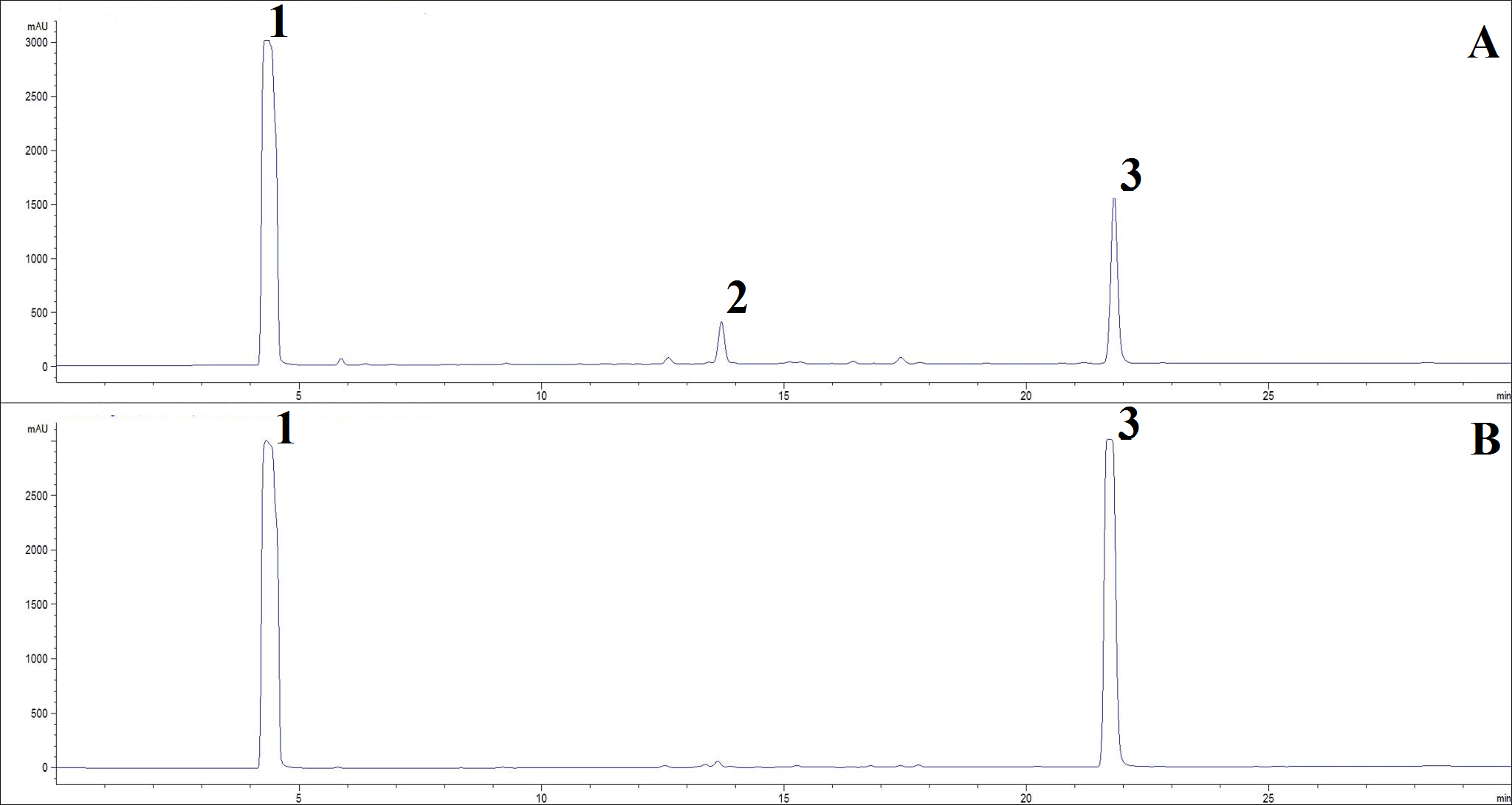

Supplement: Supplementary file 1 — Chromatogram of GA supernatant dissolved in methanol. (PNG 173 kb) [file 12906_2019_2631_MOESM1_ESM.png]

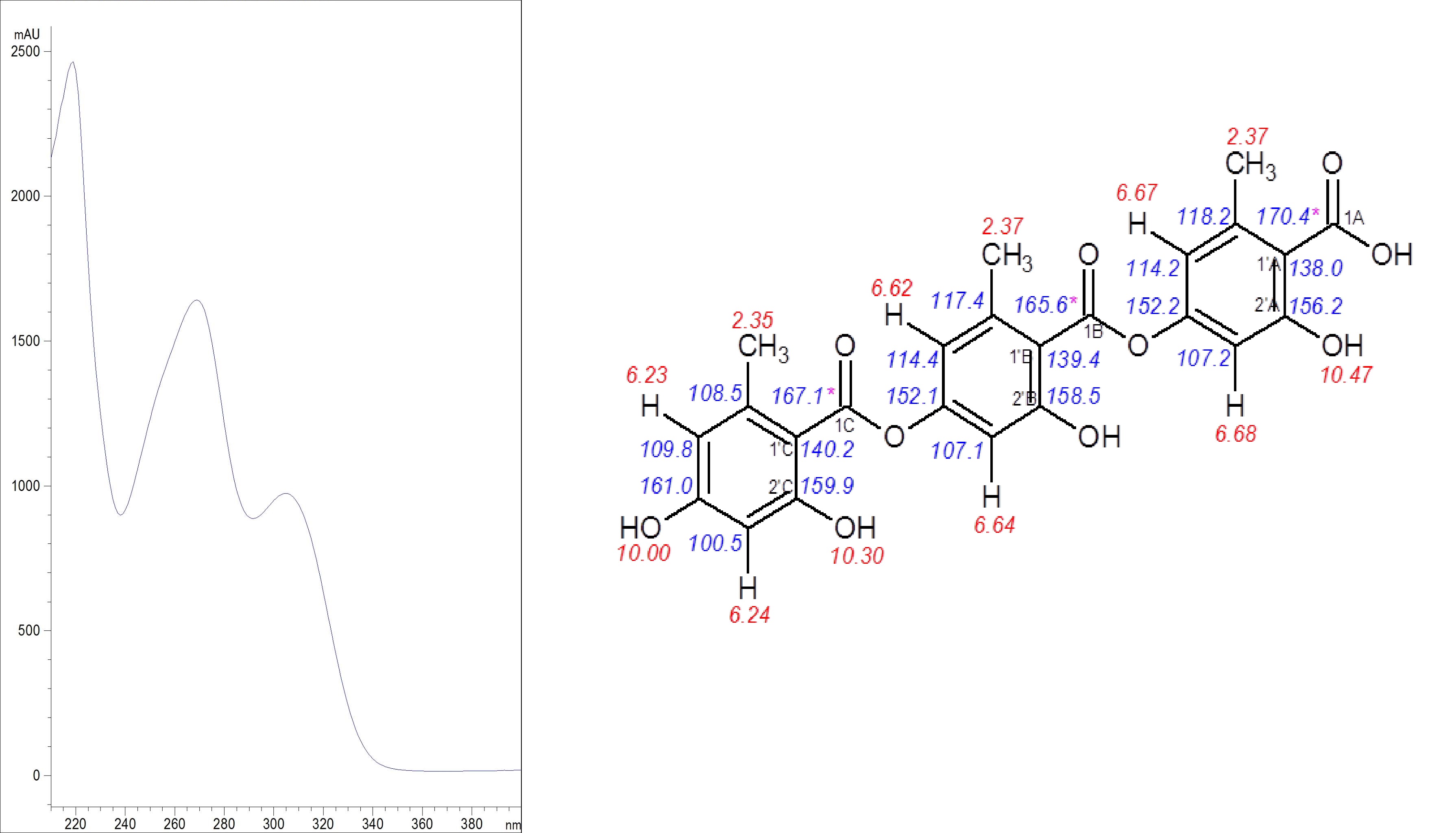

Supplement: Supplementary file 2 — UV-spectrum from HPLC and the structure of gyrophoric acid. (JPG 463 kb) [file 12906_2019_2631_MOESM2_ESM.jpg]

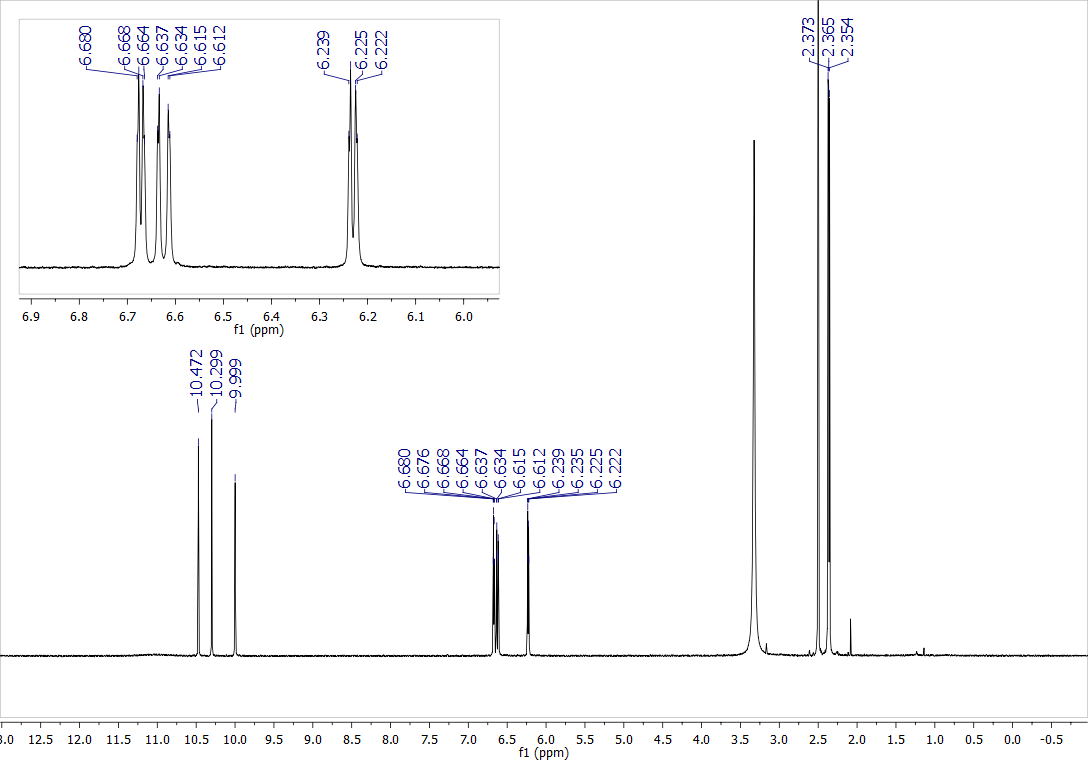

Supplement: Supplementary file 3 — 1H-NMR Spectrum of gyrophoric acid from acetone extract of the lichen Umbilicaria hirsuta. (BMP 2422 kb) [file 12906_2019_2631_MOESM3_ESM.bmp]

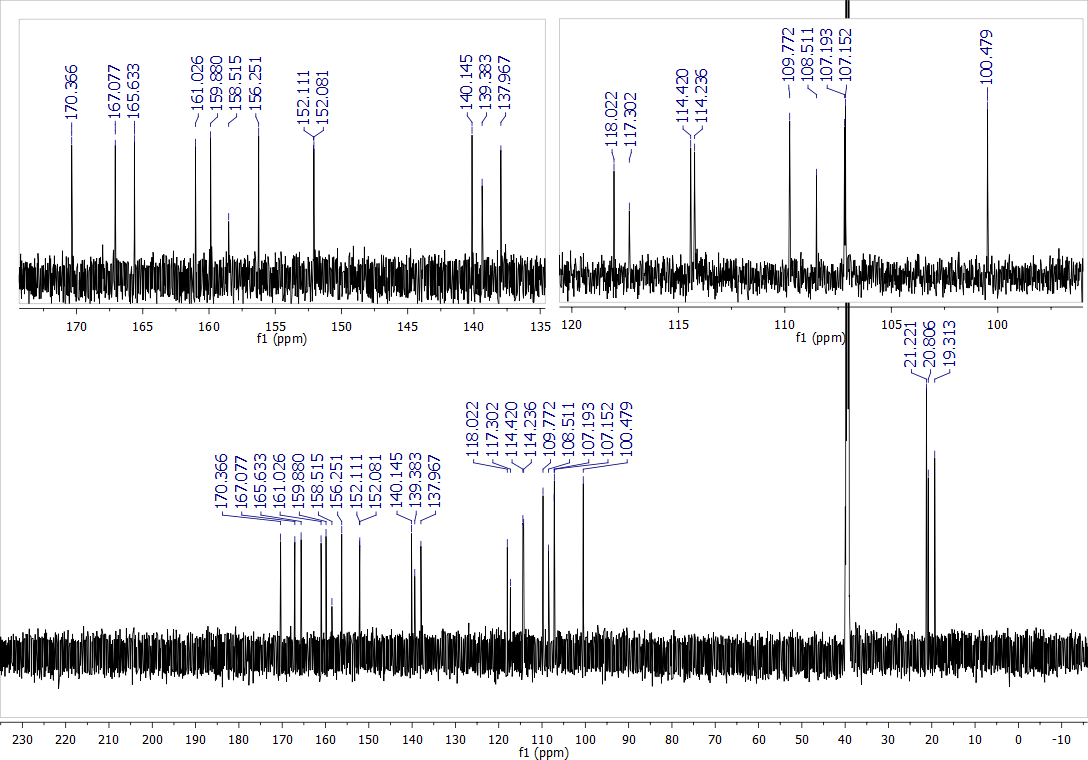

Supplement: Supplementary file 4 — 13C-NMR Spectrum of gyrophoric acid from acetone extract of the lichen Umbilicaria hirsuta. (BMP 2422 kb) [file 12906_2019_2631_MOESM4_ESM.bmp]

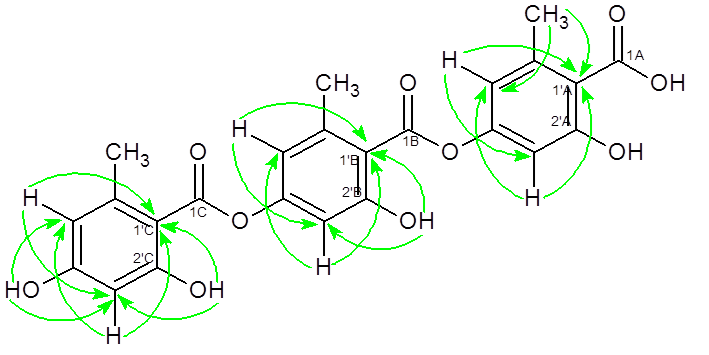

Supplement: Supplementary file 5 — Selected HMBC correlations in gyrophoric acid. (PNG 16 kb) [file 12906_2019_2631_MOESM5_ESM.png]

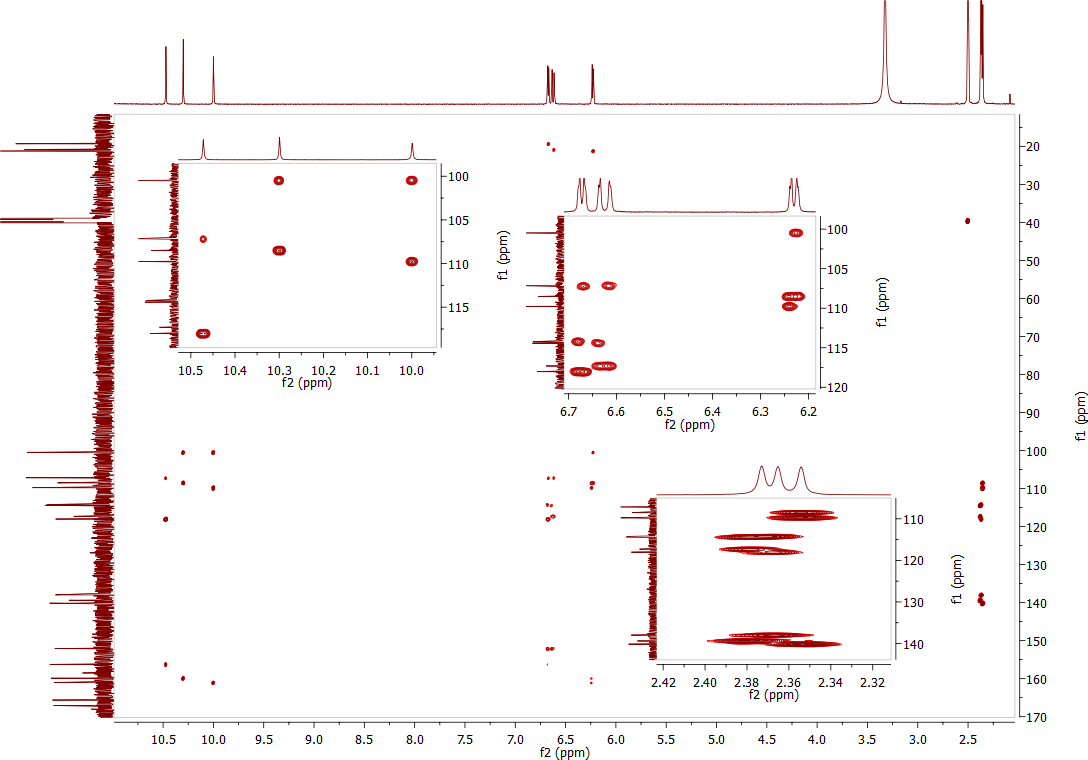

Supplement: Supplementary file 6 — HSQC spectrum of gyrophoric acid (BMP 2422 kb) [file 12906_2019_2631_MOESM6_ESM.bmp]
